# A norm about harvest division is maintained by a desire to follow tradition, not by social policing

**DOI:** 10.1073/pnas.2413214122

**Published:** 2025-06-20

**Authors:** Minhua Yan, Zhizhong Li, Yuanmei Li, Robert Boyd, Sarah Mathew

**Affiliations:** ^a^Institute for Advanced Study in Toulouse, Toulouse School of Economics, University of Toulouse Capitole, Toulouse 31080, France; ^b^Department of Human Behavior, Ecology and Culture, Max Planck Institute for Evolutionary Anthropology, Leipzig 04103, Germany; ^c^Derung norm dynamics project Dizhengdang division, Gongshan, Yunnan 673503, China; ^d^School of Human Evolution and Social Change, Arizona State University, Tempe, AZ 85281; ^e^Institute of Human Origins, Arizona State University, Tempe, AZ 85281

**Keywords:** social norms, decision-making, conformity bias, norm maintenance

## Abstract

Determining how people behave in contexts governed by social norms can clarify both how norms influence human behavior and how norms evolve. We examined cooperative farming harvest division among the Derung, a Tibeto-Burman-speaking horticultural society in southwestern China. In the village of Dizhengdang, the norm dictates that cofarming harvests should be divided equally among participating households. This contrasts with an alternative norm followed in some other Derung villages that holds that harvests should be divided equally among participating laborers. Rational choice theory and evolutionary models of norm-based cooperation assume that individuals weigh the material and social payoffs of different actions and follow norms because doing so maximizes their payoff. However, the behavior of the Derung in Dizhengdang is not consistent with payoff maximization. Using interviews on co-farming behaviors and attitudes, along with an ultimatum game experiment framed as co-farming harvest division, we found that most respondents preferred divisions based on labor contribution. They also accurately guessed that others shared this preference and would approve of such divisions. Nonetheless, they still followed the prevailing norm of dividing by household. Their self-reported explanation for this behavior was that they desired to follow their traditional practices. Such a normative decision-making algorithm can allow individually consequential norms to persist without costly policing by other group members.

## Significance

Many researchers and policymakers believe that people comply with norms to avoid negative social payoffs. However, data from the Derung people in southern China suggest that they adhere to a harvest division norm primarily out of a desire to follow tradition, even though they dislike the norm and do not expect coordination failures or social sanctions for deviating from it. This suggests that norms can be maintained without costly policing by other group members and that incentive-based policy interventions may be less effective than expected in driving norm change.

Social norms—common behaviors within a population and widespread beliefs and expectations that support them—are powerful drivers of human social life and a key source of variation between human cultural groups. Social norms can motivate people to behave in ways that do not align with their personal values or material incentives (e.g., refs. 1 and 2). Both classical rational choice theory and evolutionary models of social behavior assume that people follow social norms to prevent negative consequences from social sanctions or coordination failures (3–9). This payoff-based framework is supported by empirical work (e.g., ref. 10) and has been widely used to understand norm maintenance (e.g., refs. 11–13) and to engineer norm change (e.g., refs. 14–16).

While economists and evolutionary social scientists have often focused on payoff considerations, researchers in other disciplines have emphasized psychological biases such as conformity bias, status quo bias, ingroup bias, and moral preferences. Information that others behave in a certain way motivates people to do the same (17–22). Default or previously chosen options seem more appealing and receive more support (23, 24). Certain practices become identity markers, leading people to feel a sense of belonging when they adhere to them (25). When practices are moralized, people derive psychological payoffs from doing the right thing (26). Potentially due to these psychological biases, information about what others do (descriptive norms) often has a stronger influence on normative behavior than information about what others believe one should do (injunctive norms) (27, 28), but see refs. 29 and 30). These psychological biases may have been favored through human evolution if, on average, they reduced decision-making costs, facilitated the acquisition of locally relevant information, or encouraged compliance with socially enforced norms. To incorporate these biases, rational choice theory is sometimes modified to include a norm-compliance utility (31–36). In this paper, we use the term “psychological biases” to encompass all these mechanisms.

Determining the relative influence of payoff considerations and psychological biases on normative behavior is key to understanding norm maintenance and norm change. For example, if decisions about female genital cutting are primarily driven by payoff considerations, then severe legal punishments, information campaigns about its health costs, equal marital prospects for uncut girls, and evidence that families with uncut girls do not face reputation loss or social exclusion should lead to abandonment of the practice. In contrast, if the decisions are driven by conformity bias, status quo bias, ingroup bias, or moral preferences, the practice may persist despite these efforts. In such cases, abolishing it would require coordinated community-wide changes in behavior and beliefs. Detailed empirical studies on how people make normative decisions can determine the relative influence of payoff considerations and psychological biases in different contexts. This, in turn, can clarify the forces that sustain norms in the real world and improve our ability to predict when and how norms will change.

In this paper, we describe how individuals in a small-scale Tibeto-Burman-speaking society, the Derung, make decisions about how to divide the harvest from multihousehold subsistence crop cofarming. Traditionally, some Derung groups divided the harvest equally by household regardless of how much land or labor each household had contributed. However, recent market integration has decreased the ecological payoff of this norm compared to divisions that reward labor and land contribution. This change provides an opportunity to assess the relative importance of expectations about material and social payoffs versus psychological biases favoring established norms in Derung normative decision-making. Using data from interviews on cofarming behaviors and attitudes, and an ultimatum game framed as cofarming harvest division, we show that Derung respondents expect labor-based divisions to yield higher material payoffs without incurring social costs. If payoff considerations were driving their normative behavior, the Derung should be expected to divide the harvest by labor. Instead, most Derung people divide equally by household, stating a desire to follow tradition and align with common practice.

Our findings demonstrate that a materially consequential norm among the Derung is maintained by psychological biases, not by payoff considerations such as consequences of miscoordination or social sanctions. Because these psychological biases do not entail costs associated with monitoring others’ behavior or administering social sanctions, they circumvent the second-order free-rider problem and expand the conditions under which norm-based cooperation could have evolved in humans.

## Ethnographic Context

The Derung are a Tibeto-Burman-speaking population living in Yunnan, China, and Kachin, Myanmar. In Myanmar, they are known as the Rawang. In China, approximately 7,000 Derung people live in six villages spread along the Derung river between the Gaoligong mountain and the Dandanglika mountain in Gongshan Derung and Nu Autonomous County (see the map in *SI Appendix*, Appendix A). Historically, upstream and downstream Derung villages in China were exposed to different political influences and had little contact with each other. As a result, these two regions maintained different social norms and dialects. Our study was conducted in car-accessible settlements in the most upstream Derung village, Dizhengdang. The settlements comprised 112 households and approximately 450 villagers.

Traditionally, Derung people farmed subsistence crops on swiddens. Cooperative farming, or cofarming for short, occurs when multiple households work together to produce agricultural products but do not consume the harvests together (for subsistence crops) or deposit their money in one common pool (for cash crops). Cofarming differs from share-cropping in that all participants work together. A land owner who provides the land but does not participate in the labor does not count as a cofarming participant. Cofarming also differs from helping, as households who participate in cofarming are entitled to a share of the harvests. If the reward for a participant’s labor work is casual (often for subsistence crop farming) or independent of the harvest (often for cash crop farming), they are considered as a helper or a hired laborer, but not a cofarming participant.

Historically, in upstream Derung settlements, including those in Dizhengdang, horticultural harvests were divided equally among participating households (“household division” or “HD”) even when the households contributed different numbers of laborers. In contrast, in downstream Derung settlements, harvests were divided equally among frequently attending laborers (“labor division” or “LD”). In both upstream and downstream settlements, a cofarming participant who provided the land did not receive more, but participants who provided more seeds were allocated a larger share.

Chinese Derung villagers have experienced significant political, economic, and ecological changes in recent years. In 2002, the “sloping land conversion” contract between the Chinese Derung and the Chinese government took effect (37). This contract mandated that farmland on mountain slopes steeper than 25 degrees be converted back into forest. Between 2002 and 2005, 97.2% of the total farmland in Derung town was converted (37). As compensation, each Derung individual received 374 Jin (equal to 187 kg) of rice per year from 2003 to 2016 (38). Beginning in 2017, households were instead compensated with cash based on the amount of land they relinquished. In 2006, Dizhengdang was connected to China’s national road network. In 2014, a tunnel replaced a section of the road that was blocked by snow during wintertime, granting Chinese Derung people year-round access to the market. Also in 2014, the government built modern houses for the Derung. As a consequence, some Dizhengdang villagers lost part of their farmland, and some now live far from their land, and they rely on cofarming with others to access more farmland or farmland in convenient locations. These changes have increased the value of land and labor.

For Dizhengdang villagers, the culturally specified ideal arrangement for subsistence crop cofarming is as follows: each participating household contributes the same number of frequently attending laborers; they schedule days when these laborers will be available to work on the cofarmed land together; if additional laborers from any household are available on a work day they may also join as helpers; on a work day, all laborers gather at the kitchen of one household to eat breakfast and then go to the land together; they take breaks together to eat, drink, or rest and resume work at the same time; at the end of the day all laborers stop working at the same time and go home together. Dizhengdang villagers frequently cofarm even when they cannot achieve the ideal arrangement. Households with different numbers of laborers can cofarm, and additional laborers from a household may attend every day, but are considered “helpers.” When some or all laborers from a household cannot attend due to illness, caretaking responsibilities, government work, etc., the other households may work on their own, and the absent party does not need to make up for the missed work.

## Study Design

To study how norms influence behavior, Bicchieri (39) has proposed measuring three kinds of belief: 1) empirical expectations, what the person thinks people who matter to them would do in the same situation, 2) normative beliefs, what they think people should do; and 3) normative expectations, what they think people who matter to them would think people should do. A payoff-maximizing individual estimates the social payoffs of different behavioral options based on their empirical and normative expectations, weighs these against the material payoffs, and adopts the option with the highest net payoff as their normative behavior. Our study was designed based on this framework.

We identified the individual who made decisions about farming and harvest division in each household in Dizhengdang, referred to from now on as “deciders.” Following Bicchieri’s framework (39), we measured deciders’ normative behavior, normative beliefs, and normative expectations. We did not measure their empirical expectations because it was common knowledge among Dizhengdang villagers that household division was the prevailing practice. The normative behavior, beliefs, and expectations were measured through four research activities: 1) general surveys, 2) semistructured interviews on real-life cofarming partnerships, 3) an ultimatum game framed as cofarming harvest division, and 4) postgame surveys. The design and implementation details of these research activities are provided in *SI Appendix*, Appendix C through *SI Appendix*, Appendix E.

In the general surveys, we measured deciders’ normative beliefs by asking them to ignore the current practice and choose a division rule for the village. In the semistructured interviews, we assessed deciders’ real life normative behavior by asking them to recall their own and their partners’ labor and land contribution, each party’s division proposals, and the eventual realized division. To measure normative expectations, we asked deciders how they believed their partner(s) and other villagers would react if they had raised different division proposals. To measure social sanctions they would exert on others who deviated from the norm, we asked deciders how they would react if their partner(s) had raised different division proposals.

Additionally, we conducted an ultimatum game to assess deciders’ normative behavior, beliefs, and expectations in a controlled setting. In an ultimatum game, a proposer proposes how to divide a resource between themself and a paired responder, and the responder either accepts or rejects the division proposal. If the responder accepts, both parties receive the amount of resource specified in the proposal. If the responder rejects, both parties receive nothing. The ultimatum game closely mirrors cofarming harvest division: Participants divide a resource; the responder decides whether to accept a division proposal; a rejection of the division proposal may end the cofarming partnership, denying both parties of its benefits; and the proposer must consider the responder’s potential reaction when deciding what to propose. This experimental setting allowed us to manipulate the relative contribution of cofarming households by randomly assigning participants into one of two treatments, one where the proposer and responder contributed the same number of laborers and another where the proposer contributed twice as much labor as the responder. It also allowed us to control for the relationship between cofarming partners by randomly pairing proposers and responders and keeping their identities anonymous. In addition, the ultimatum game directly measured deciders’ normative behavior. To elicit their normative beliefs and normative expectations within the ultimatum game setting, we conducted postgame surveys and asked deciders to evaluate different proposals in both treatments and to guess how others would evaluate them.

## Results

### Household Division Is the Most Common Behavior in Real Life and the Ultimatum Game.

**Fig. 1.**
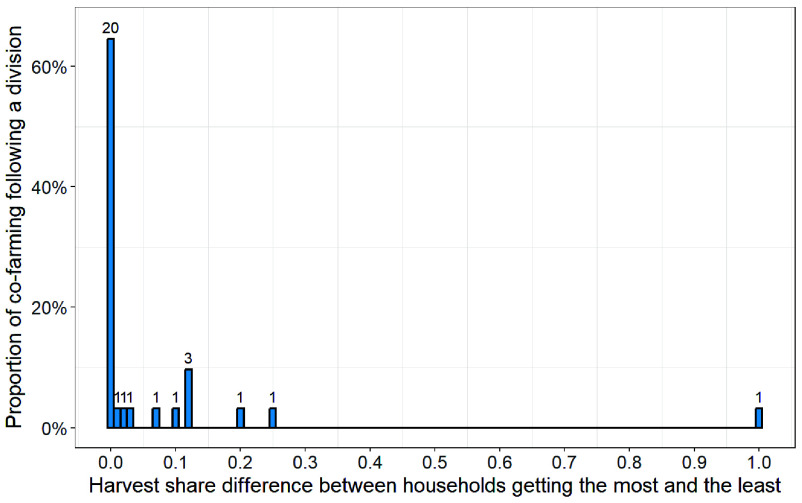
Reported divisions in real-life cofarming, with division quantified as the share difference between the household receiving the largest portion of the harvest and the household receiving the smallest portion. A value of 0.0 on the x-axis represents equal division by household (HD). HD is the most common reported division in real-life cofarming.

We measured real-life normative behavior by analyzing the distribution of divisions in a sample of 47 cofarming partnerships reported by 60 interviewees from 60 different households. Among the 47 partnerships, 31 underwent a harvest division process (see *SI Appendix*, Appendix C.2 for why the others did not). Fig. 1 presents the distribution of divisions in these 31 partnerships, with division quantified as the share difference between the household receiving the largest portion and the household receiving the smallest. A value of 0.0 thus represents equal division by household (HD), while 1.0 indicates that all the harvest was allocated to a single household. According to deciders’ reports, HD is the most common division in real-life subsistence cofarming, occurring in 20 out of 31 cases in the sample. We preferentially sampled non-HD divisions (see *SI Appendix*, Appendix C.2 for the sampling criteria), so the dominance of HD in our results occurs despite this bias.

**Fig. 2.**
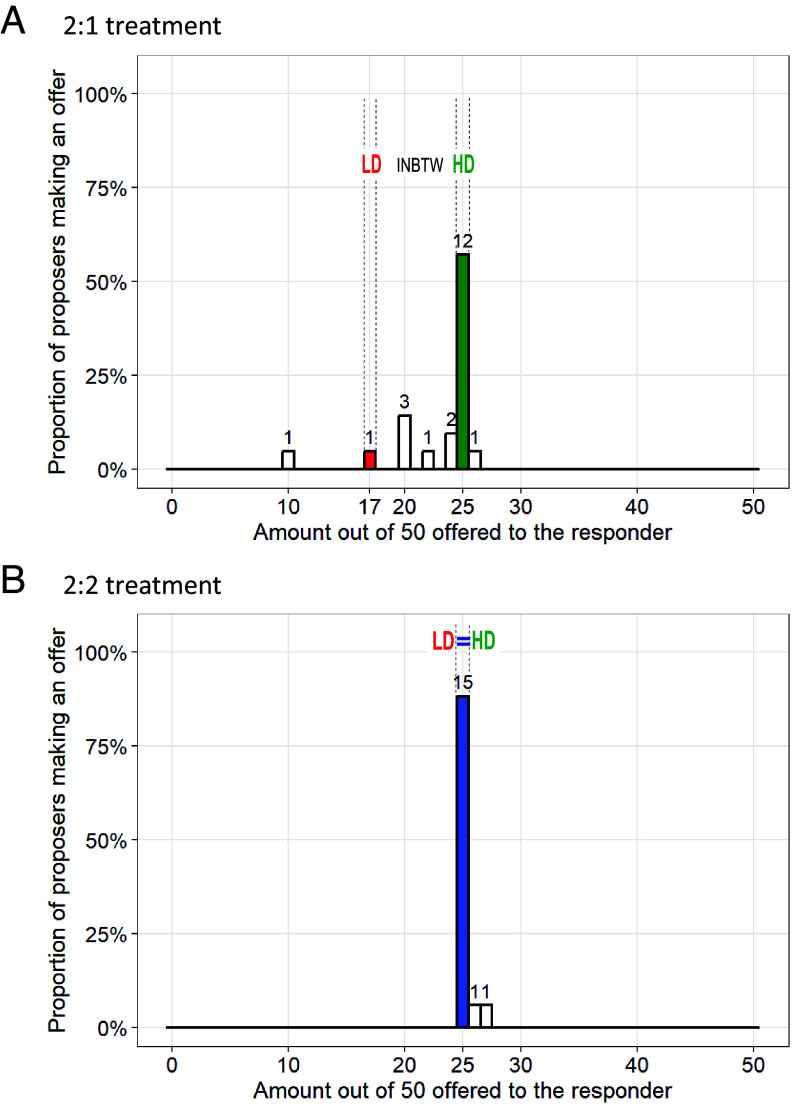
Distribution of Ultimatum Game Offers. (*A*): In the 2:1 treatment, most offers followed household division (HD). In this treatment, the proposer household had two residents and contributed two laborers, while the responder household had only one resident and contributed one laborer. Although the needs and labor contributions of the households clearly differed, most Derung proposers (57%) divided equally by household, offering half the total harvest (25 out of the 50 Jin of rice) to their paired responders. Others rewarded needs and labor contributions to different degrees, and one proposer offered more than HD to the responder. (*B*): In the 2:2 treatment, most offers were half the harvest, i.e., 25 out of the 50 Jin of rice. Here, both the proposer household and the responder household had two residents and contributed two laborers. Since labor contributions were equal, labor division and household division coincided and half the harvest was the most common (88%) offer.

Deciders’ recollections of real-life cofarming divisions may have been biased toward HD, as HD was the norm and was cognitively salient. In contrast, in the ultimatum game normative behavior was measured directly as offers made by proposers. The ultimatum game had two treatments: in the 2:2 treatment, the proposer and responder households each had two residents and contributed two laborers; in the 2:1 treatment, the proposer household had two residents and contributed two laborers, while the responder household had one resident and contributed one laborer. In both treatments, participants were told that the cofarming yielded 50 Jin (equal to 25 kg) of rice as the harvest, and a proposer was asked to divide it between themself and their partner, the responder. Even in the 2:1 treatment, most proposers offered their responder 25 out of the 50 Jin of rice, the division consistent with HD (Fig. 2).

### People’s Normative Behavior in Real Life and the Ultimatum Game Does Not Stem from Their Normative Beliefs.

Although household division is the most common division in practice, the majority of deciders prefer division according to labor contribution, indicating that normative behavior and normative belief are not aligned. When asked to choose a harvest division norm for the whole village to follow from then on, 19 out of the 33 deciders who participated in the general survey chose division in proportion to labor contribution (“LD”); 13 chose HD and one chose giving the household that contributed more laborers somewhat more but not as much as in proportion to labor contribution (henceforth “INBTW” for being in-between LD and HD). The higher incidence of “HD” in real-life cofarming divisions compared to its incidence as the preferred village norm is statistically significant with P=0.042 (*SI Appendix*, Appendix F.1).

Data from the ultimatum game further illustrate the discrepancy between normative behavior and beliefs. Among the 21 proposers in the 2:1 treatment, 13 made “HD” offers or offered even more to the responder household with fewer laborers, yet only 4 of these 13 proposers believed HD was the best division. Two of the 21 proposers believed “INBTW” was the best offer and proposed “INBTW” offers. The majority (15 out of 21) of the 2:1 treatment proposers preferred “LD,” i.e., dividing in proportion to the number of laborers, but these 15 proposers gave various offers, including offers that overcompensated labor contribution, LD, INBTW, and HD, with HD being the most common, offered by 9 proposers. There is only a weak correlation between how a proposer divided in the 2:1 treatment and what they stated was their preferred division, with Spearman’s ρ= 0.26 (*SI Appendix*, Appendix F.2).

Additionally, offers in the 2:1 treatment did not align with the population-level normative beliefs, as estimated by the preferred division of all postgame survey respondents. The postgame survey recruited 72 of the 74 participants from the ultimatum game. When asked to suggest the best offer in the 2:1 treatment, only 13 out of the 72 respondents suggested HD; 52 suggested LD, 4 suggested INBTW offers, and 3 suggested best offers that overcompensated the household with more laborers, potentially because these 3 respondents aimed for labor division, (17/33 for responder/proposer), but adjusted it to 15/35 or 10/40 for ease of implementation. The incidence of “HD” in the 2:1 treatment offers is significantly higher than in the preferred division reported by the 72 postgame survey respondents (P=0.00008) (*SI Appendix*, Appendix F.3).

### Proposer Behavior in the Ultimatum Game Does Not Stem from Inaccurate Normative Expectations.

Even if most people dislike HD, it could persist due to pluralistic ignorance (40–42). According to this hypothesis, people do not like HD but continue to follow it to avoid sanctions, because they incorrectly infer from others’ behavior that HD is widely supported and believe that they would be punished if they expressed their true belief that labor-based divisions are better. In other words, their norm compliance stems from inaccurate normative expectations. However, pluralistic ignorance does not explain why HD was the most common offer in the ultimatum game 2:1 treatment. The postgame survey incentivized the 72 respondents to guess the majority preferred division, i.e., what division most other people in the village would think the best division was (*SI Appendix*, Appendix E.2). Most respondents (51 out of 72) correctly guessed that the majority of other villagers would prefer labor division in the 2:1 treatment, while 16 guessed HD, 4 guessed an INBTW division and 1 guessed a division that overcompensated the proposer’s labor contributions.

### People form Normative Expectations by Projecting Their Normative Beliefs on Others.

We fit a Bayesian model (*SI Appendix*, Appendix G) to predict how a respondent guessed the majority preferred division based on their own and their real-life cofarming partners’ preferred divisions reported in the postgame survey. The model indicates that Derung deciders primarily projected their own preferred divisions onto others, rather than assuming that those who followed HD did so because they genuinely believed it was the best division. Additionally, the respondents did not base their guesses on their cofarming partners’ preferred divisions. The basis of the respondent’s guess could impact their confidence in its accuracy. Specifically, a respondent may expect their normative expectations to be less accurate if they guessed solely based on their own preferred division compared to if they knew and considered their cofarming partners’ preferred divisions, a more direct form of information about the majority normative beliefs. Such uncertainty about others’ beliefs is common during cultural change (e.g., ref. 43) and may explain why Derung proposers in the 2:1 treatment did not divide by labor, despite accurately expecting that most others would prefer it.

### Deviations from HD Do Not Lead to Social Disapproval in Real-Life Cofarming.

Social disapproval is also not the reason why HD persists in Derung cofarming. In interviews about real-life cofarming partnerships, deciders rarely expected second-party or third-party disapproval. Twenty-four respondents who had contributed more labor, land, or seeds in their real-life cofarming partnerships were asked to predict how their partner(s) would react if they proposed to give themselves more than HD. Among the 24 respondents, 7 stated they would not make such a proposal and refused to speculate on the partners’ reaction; 11 predicted that the partner(s) would accept the proposal, would not be upset, and would not discontinue the cofarming; 4 predicted that the partner(s) would either reject the proposal and/or feel upset and/or discontinue the cofarming; 2 said they could not guess how their partner(s) would react.

The anticipated reactions of respondents to hypothetical selfish non-HD proposals from their partners show the same pattern. Seventeen respondents were asked how they would react to division proposals in which the partners gave themselves more. Among the 17 respondents, 15 answered that they would accept the proposal, would not feel upset, and would not discontinue the cofarming; one respondent was unsure how they would react; and one respondent said they would agree to give the partner household more and would not feel upset, but would decide whether to discontinue the cofarming depending on whether the partner was requesting more due to need or as a way to indicate their dissatisfaction about the cofarming arrangements. As for third-party attitudes toward a person proposing labor division, most respondents (57 out of 60) either did not know how others would react or believed that others would not care. One respondent expected others to approve, while two expected others to view the person as calculating and laugh-worthy. Taken together, these findings indicate that deciders correctly expect second-party and third-party disapproval to be infrequent, and mild if it occurs at all. Therefore, social costs for deviations are not the primary reason why most Derung deciders follow HD in real-life cofarming.

### Labor Division Is as Frequently Accepted as Household Division in the Ultimatum Game.

**Fig. 3.**
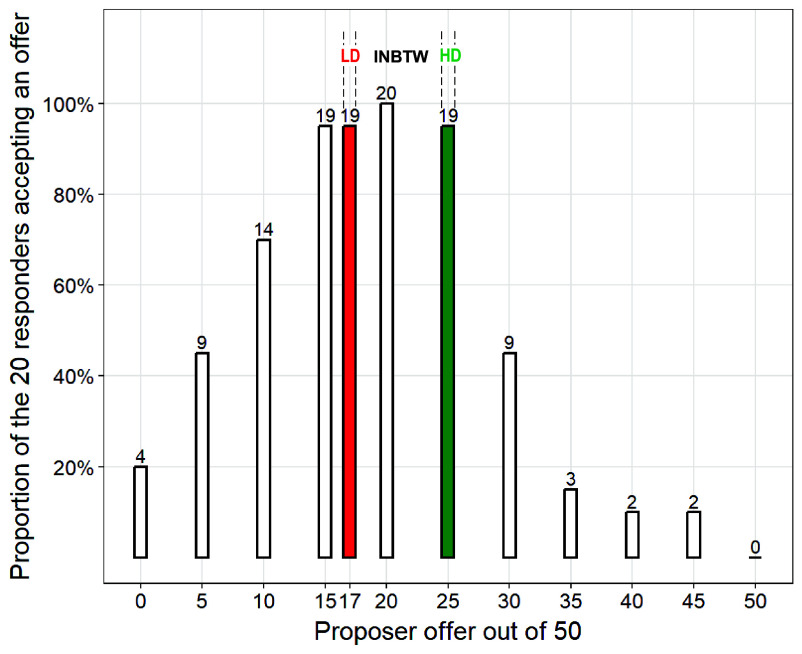
Ultimatum Game 2:1 Treatment Responders’ Acceptance Rate of Different Offers. A proposer in the 2:1 treatment could make offers consistent with labor division (LD) and still achieve the same level of acceptance as offering household division (HD). Each of the 20 responders in the 2:1 treatment was asked whether they would accept the offer if it was 0, 5, 10, 15, 17, 20, 25, 30, 35, 40, 45, or 50 out of the total harvest of 50 Jin of rice. If the proposer followed LD, their offer would be 17 out of 50, while HD corresponded to an offer of 25. One responder, who only participated in cash crop cofarming in real life, did not accept HD. All 20 responders accepted the offer of 20 Jin to themselves and 30 Jin to the proposer household who contributed twice as much labor—an offer that fell between LD and HD. One responder, who had only become a decider shortly before the ultimatum game, did not accept LD.

Although ultimatum game proposers in the 2:1 treatment preferred labor division and expected most others to prefer labor division, they may have chosen to divide equally by household because they feared that the responders would reject lower offers due to the norm. However, responder behavior suggests otherwise. A proposer in the 2:1 treatment could make offers consistent with labor division and still achieve the same level of acceptance as offering HD (Fig. 3).

Did proposers know that responders would almost always accept labor division? No data directly address this question. However, it is widely acknowledged among Dizhengdang villagers that maintaining harmony is the primary goal in social interactions and that rejecting a fellow villager’s request would be awkward. Additionally, no proposer cited fear of non-HD offers being rejected as a motivation for offering HD. Therefore, in the ultimatum game, considerations of second-party reactions were, at most, subconscious.

### HD Is the Default Normative Behavior in Real-Life Cofarming Because It Is Tradition and Common Practice.

A cofarming partnership can arrive at HD through different processes. Among the 20 partnerships that followed HD, 15 implemented it as the default without any discussion about how to divide the harvest. In two partnerships, HD was the only division proposed and all partners accepted it. In the remaining three cases, one participant initially proposed a non-HD division that would have allocated more to their partner(s), but the partner(s) rejected the proposal and counterproposed HD, which was then accepted and implemented. Among the 11 cofarming partnerships that followed non-HD divisions, six involved one party proposing a division that allocated more than HD to their partner(s), which the partner(s) directly accepted. In one case, both participants proposed giving the household that contributed more labor a share greater than HD but smaller than LD. In four cases, one party implemented a division that allocated more than HD to their partner(s), either before the partner(s) could object or despite the partner(s)’ objections. Therefore, HD is often followed as the default in real-life cofarming, while non-HD divisions require explicit discussions and sometimes forcible implementations. Forcible implementations can involve placing additional harvest into the cofarming partner’s basket despite their objection, or leaving part of one’s harvest share on the cofarmed land and insisting that the cofarming partner take it.

**Fig. 4.**
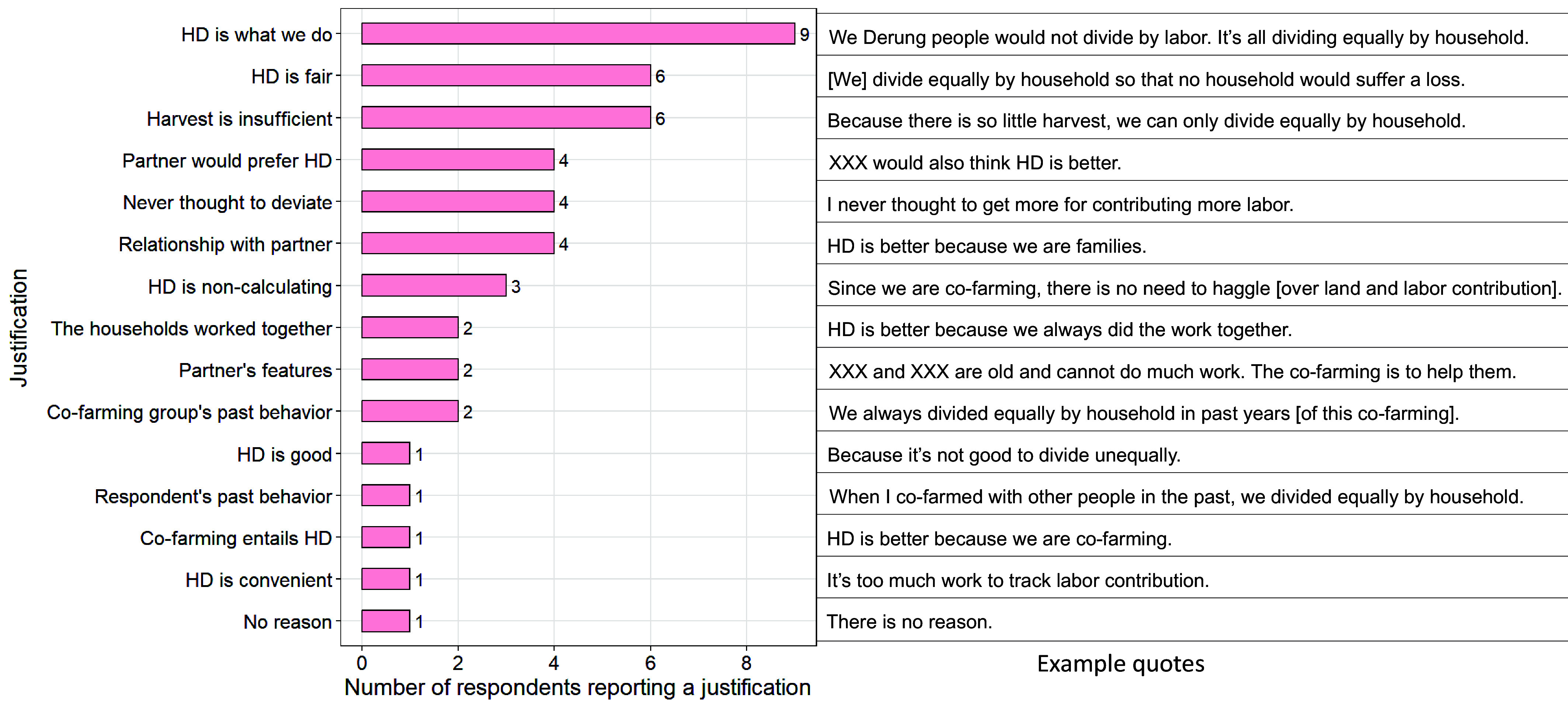
Distribution of Respondents’ Justifications for Choosing Household Division (HD) and Example Quotes. The most frequently reported justification was that “HD is what we do.”

HD is followed as the default because it is tradition and common practice. For each cofarming partnership in which they participated, respondents reported how they would divide the harvest if their partner(s) asked them to make the decision, and offered justifications for their division decisions. When a respondent stated they would divide equally by household, the most frequently cited justification was that “HD is what we do” (Fig. 4). Three other justifications were closely related—1) “cofarming group’s past behavior:” The respondent and their partners in the coded cofarming had followed HD when they cofarmed in the past, 2) “respondent’s past behavior:” the respondent and their other cofarming partners had followed HD in the past, and 3) “cofarming entails HD:” HD was the natural division arrangement for cofarming. Moral justifications were the second most common and included “HD is fair,” “HD is noncalculating” and “HD is good.” The third most common type of justification referred to specifics of the cofarming and included “harvest is insufficient,” “the households worked together” and “partners’ features” such as the partners being elders. Other justifications included: normative expectations—the respondents believed that their partners would prefer HD; cognitive justifications—the respondents never thought to deviate from HD; kinship or friendship with the partners; and practicality—HD was convenient because it did not require tracking labor. In addition, one respondent stated that there was no reason why they chose HD.

### Normative Behavior in the Ultimatum Game Was Driven by a Desire to Follow Tradition.

In real-life cofarming, choosing to follow HD even when it is disadvantageous could signal care for one’s partner and a commitment to the partnership. This social payoff, combined with the belief that households contributing more should have greater influence over the division, may explain why HD was often followed in real-life cofarming despite being the less preferred general division rule. However, this motivation was absent in the ultimatum game, since the participants were anonymous. In the 2:1 treatment, there were eight (out of 21) proposers who reported preferring labor division and expecting most others to prefer labor division, but followed HD behaviorally and offered 25 out of the total harvest of 50 Jin of rice. When asked why, all eight respondents indicated a motivation to follow tradition. One respondent captured this sentiment well, stating: “What people think is just what people think. When we do things, we do what we have always been doing.”

## Discussion

### The Household Division Norm Does Not Persist Due to Personal Normative Belief, Pluralistic Ignorance, or Social Policing.

Our findings are inconsistent with many accounts of how social norms are maintained. Some researchers believe that a norm can persist because it aligns with people’s normative beliefs about what is right or good. Normative beliefs may be shaped through internalization such that people receive a psychological payoff from norm compliance (44–48), or the norm may initially arise from self-interest and later become moralized as the “right” and “good” way to act (49, 50). However, our findings show that while HD is the most common behavior in both real-life cofarming and an economic game, most Derung individuals prefer labor-based divisions (LD) instead. To explain why a disliked norm can persist, some researchers emphasize the role of mistaken beliefs about others’ normative beliefs. However, in our study, most participants have accurately predicted that most others in their village would also prefer labor division. Another hypothesis suggests that people follow norms that are otherwise not in their private interest because deviating from them elicits social sanctions. However, our data suggest that individuals who deviate from HD do not face social costs. People generally approve of non-HD division proposals, and they expect others to do the same. In the ultimatum game, LD offers were accepted at the same high rate as HD offers. Instead of personal normative beliefs, pluralistic ignorance, or social policing, participants in our study follow the household division norm because it is what they have always been doing. Therefore, Derung people’s behavior in a consequential resource division context is guided by a descriptive norm rather than the injunctive norm.

Our findings align with many previous studies conducted in WEIRD societies. In a dictator game, people ignored the injunctive norm and followed the descriptive norm when the two conflicted (27). Similar behavioral patterns have been observed in studies on food choice (51, 52), alcohol use (53–55), energy and resource conservation (56, 57), tax paying (58), corruption (59), and mask wearing and social distancing during the COVID-19 pandemic (28, 60, 61).

### The Fact That Household Division Is Equality-Based Does Not Fully Explain Its Persistence.

Equal division rules, such as the household division norm followed by Dizhendang cofarmers, are widely practiced in cooperative production across societies (11, 62–64) due to their psychological and dynamical advantages over alternative division rules. First, equal division rules align with inequality aversion preferences. Western participants often donate 50% in dictator game experiments (65) and dislike both disadvantageous and advantageous inequality (66–70). Second, equal division serves as a focal point, facilitating coordination when no norm exists and communication is unavailable (71, 72). Repeated adoption of this salient solution may then establish it as a norm. Third, equal division is more dynamically stable. It is the unique stable bargaining solution when bargaining roles can reverse (73) and is more resistant to mistakes and experimentation (74). Additionally, equal division rules leave less room for participants in cooperative production to manipulate their own contributions or misinterpret those of others, thereby reducing perception errors that could corrode cooperation (75). These benefits may outweigh the costs of reduced incentives and lower individual contributions to the joint effort.

These advantages could explain why the HD norm can resist invasion by some alternative norms—it ensures equality, is more salient, and is less ambiguous. For example, compared to tracking labor contributions and dividing accordingly, HD is more convenient, establishes clear expectations about payoffs from cofarming even though unforeseeable events can affect labor contributions, and avoids disputes over the accuracy of labor tracking.

While these advantages may have contributed to the establishment and persistence of the HD norm, they are not consciously expressed in the decision-making of Dizhengdang villagers. Only one respondent cited “HD is convenient” as the motivation for following HD in real-life cofarming. In addition, Z.L., Y.L., and three other Derung fieldworkers documented the daily activities of 65 households throughout the farming cycles in 2020 and asked participants whether they wanted to know their own and their partners’ labor contributions in cofarming. Among the 65 households, 57 participated in one or more subsistence crop cofarming partnerships. Of these 57 deciders, 49 stated that they did not need such data, while 8 wanted it to track the total labor invested but not individual contributions.

Moreover, equal division by laborer has many of the same advantages as equal division by household. Both division rules satisfy the principle of equality, but while household division applies it at the household level, equal division by laborer applies it at the individual level. The salience of equal division by laborer is also recognized by Dizhengdang villagers, as the freely nominated best divisions in the ultimatum game are predominantly equal division by laborer. Therefore, the moral, cognitive, and dynamic advantages of equal division cannot explain why the “household as unit” interpretation of the equal division principle persists rather than being replaced by the “laborer as unit” interpretation in Dizhengdang.

### Reasons Why People Follow Descriptive Norms and Implications for Norm Change.

Why do Derung farmers “do what [they] have always been doing”? Explanations for why people base their behaviors on descriptive norms can be grouped into four categories: 1) immediate individual benefits—following the norm maximizes payoffs in the current event, 2) norm compliance as a signal—people follow norms to demonstrate desirable qualities such as impulse control, cultural knowledge and respect for rules, increasing their chances of being favored as cooperative partners in other contexts, 3) psychological payoffs—norm compliance yields psychological payoffs because it was favored by selection over the course of human evolution, and 4) cognitive heuristic—relying on established practices simplifies decision-making. Below we discuss these mechanisms in the context of our data on Derung decision-making.

People may follow the descriptive norm because the benefits of adopting a normative behavior increase with its popularity. Norms often exhibit similarity-based payoffs. Members whose behaviors are more similar to the majority receive higher payoffs because they can better coordinate with others, are evaluated more positively (76, 77), or face weaker social sanctions as social policing becomes less effective with more transgressions (78). In Derung cofarming, the established norm may serve as a coordinating device that eliminates negotiation costs and extends the range of potential partners. However, respondents did not explicitly report coordination as a motivation for following household division, and this explanation cannot account for why ultimatum game proposers followed the norm when a responder had already been assigned.

A person may gain a positive reputation and become a preferred cooperative partner by signaling their quality through norm compliance. Consistent with this mechanism, some Derung respondents saw “dividing equally by household” as indicative of a strong cooperative bond between the households. This mechanism, however, cannot explain why most proposers in the ultimatum game 2:1 treatment followed household division, as the ultimatum game was anonymous. It also cannot explain why people follow the descriptive norm rather than the injunctive norm.

A person may follow tradition because they value their group identity and have internalized the tradition as a marker of their group identity. Weak triggers of group identification have been shown to increase compliance with individually costly cooperative norms (79). Consistent with this mechanism, many Derung respondents suggested they would follow household division in real-life cofarming because it was “what the Derung do.”

A descriptive norm may become internalized as the culturally specific interpretation of cross-culturally acknowledged moral principles. Fairness and generosity are valued across many societies, but the culture specifies what is considered “fair” and “generous” in a given context. In Derung cofarming, moral justifications were the second most common justification for wanting to follow household division. Household division was considered “fair,” “good,” and “noncalculating.”

Adherence to descriptive norms may occur when general-purpose social learning strategies are applied to normative behaviors. Based on this hypothesis, people should be more likely to copy others’ normative behavior when they are uncertain about what the norm is or the social payoffs associated with different behaviors (80). This hypothesis can also explain why people value information about what others do more than what others think: Actions—rather than words—can serve as a credibility-enhancing display, and our social learning psychology has been selected to favor models with high credibility (81).

Following norms may be a cognitive heuristic. Because a norm has been followed in the past, it sets the reference point and becomes the default and most cognitively salient solution (82). Consistent with this explanation of norm compliance, household division was often followed as the default without any discussion in real-life Derung cofarming.

These different explanations of norm compliance make different predictions about how a norm will change when new payoff structures are introduced. If a person follows a descriptive norm because it maximizes similarity-based payoffs, their behavior will shift once a sufficient number of other group members adopt a new norm (83). If this is the primary motivation across the population and individuals vary in the threshold number of others who must shift before they do so, norm change may proceed as a cascade (40). On the other hand, if a person has internalized the norm as part of their group identity or moral responsibility, they are unlikely to shift even if the majority of others adopt a new practice (84, 85). In this case, norm change will occur only after the old practice is detached from identity and moral significance. Finally, if people follow the descriptive norm because it is the reference point, nudges that make a new practice more convenient than the old (86) can facilitate norm change.

### Maintaining Norms Without Costly Policing.

The finding that Derung people follow the descriptive norm in a consequential resource-allocating context despite opposing payoff considerations has important implications for how norm-based cooperation can be maintained. Social sanctions on norm violators are often costly for those who perform them: Withdrawing help results in the loss of a cooperative partner, and direct punishment may provoke retaliation (87, 88). Relying on sanctions to maintain a norm can therefore reduce group efficiency (89–91) and create a second-order free-riding problem, which requires additional mechanisms to solve (92–96).

Empirical evidence suggests that social sanctions do indeed maintain certain norms, such as participation in lethal warfare (11), and that sanctioning behavior itself is regulated by norms that encourage people to administer appropriate punishment to violators (97, 98). In contrast, some other norms can be maintained through social disapproval of mild transgressions, expressed as gossiping, mocking, or complaining (12). Social disapproval is not by itself costly to norm violators but can deter violators because it conveys the threat that more severe social sanctions may follow if transgressions continue. Compared to social sanctions, social disapproval is less effective for norm enforcement but also less costly for norm enforcers. The Derung cooperative farming division norm is not maintained by sanctions or social disapproval. Instead, it is maintained by cofarmers’ preference for conforming to tradition. This mechanism further reduces the cost of norm enforcement by eliminating the need for monitoring and expressing disapproval. People’s tendency to follow descriptive norms revealed in this and other studies suggests that normative social structures have selected for a human norm psychology that not only prompts us to comply with norms in response to social disapproval but may also motivate us to follow norms even in the absence of social pressure.

## Methods

## Materials and Methods

The data reported in this paper were collected during M.Y.’s 14 mo of stay in Dizhengdang. Z.L. and Y.L., who are residents of the study community and fluent in the Derung language, assisted M.Y. with translation and cultural contextualization of the research instruments. The general survey was conducted in 2018. The interviews were conducted in 2019 and 2020. The ultimatum game and postgame survey were conducted in 2019. These four research activities collectively covered 92 household farming deciders, 61 female and 31 male. Details on participant recruitment, the design and implementation of the research activities, as well as the statistical methods for data analysis, are provided in *SI Appendix*.

Z.L. and Y.L. communicated the general finding reported in this paper to the study community. No Dizhengdang villager raised objections regarding the findings or their interpretation.

### IRB and Human Research Ethics.

All study procedures were reviewed and approved by the Institutional Review Board (IRB) at Arizona State University. M.Y. acquired a research permit from the Gongshan County government and received verbal approval from Dizhengdang village leaders. All participants provided informed consent, with the understanding that their participation was voluntary and that they could withdraw at any time.

## Supplementary Material

Appendix 01 (PDF)

## Data Availability

Anonymized coded data of survey responses, interview answers, ultimatum game behaviors, and post-ultimatum-game survey responses associated with randomized subject IDs have been deposited in GitHub (https://github.com/Minhua-Yan/norm-tradition) (99). All other data are included in the manuscript and/or *SI Appendix*. Some study data are available: To use the data to perform additional analysis, please contact the first author M.Y. at “minhua.yan@iast.fr” or “myan18@asu.edu.” Any additional analysis must respect the cultural context, conform to the original research ethics protocol, and be disseminated to the participants.

## References

[1] B. C. Truman, The Field of Honor: Being a Complete and Comprehensive History of Duelling in All Countries; Including the Judicial Duel of Europe, the Private Duel of the Civilized World (Fords, Howard and Hulbert, 1883), vol. 1.

[2] D. Chang, R. Chen, E. Krupka, Rhetoric matters: A social norms explanation for the anomaly of framing. Games Econom. Behav. **116**, 158–178 (2019).

[3] R. Boyd, P. J. Richerson, Punishment allows the evolution of cooperation (or anything else) in sizable groups. Ethol. Sociobiol. **13**, 171–195 (1992).

[4] E. Fehr, U. Fischbacher, Social norms and human cooperation. Trends Cogn. Sci. **8**, 185–190 (2004). 15050515 10.1016/j.tics.2004.02.007

[5] J. Henrich , Costly punishment across human societies. Science **312**, 1767–1770 (2006). 16794075 10.1126/science.1127333

[6] K. Abbink, L. Gangadharan, T. Handfield, J. Thrasher, Peer punishment promotes enforcement of bad social norms. Nat. Commun. **8**, 609 (2017). 28931829 10.1038/s41467-017-00731-0 PMC5607004

[7] D. Balliet, L. B. Mulder, P. A. V. Lange, Reward, punishment, and cooperation: A meta-analysis. Psychol. Bull. **137**, 594 (2011). 21574679 10.1037/a0023489

[8] L. Balafoutas, N. Nikiforakis, B. Rockenbach, Direct and indirect punishment among strangers in the field. Proc. Natl. Acad. Sci. U.S.A. **111**, 15924–15927 (2014). 25349390 10.1073/pnas.1413170111 PMC4234623

[9] F. W. Marlowe , The ‘spiteful’ origins of human cooperation. Proc. R. Soc. Lond. B Biol. Sci. **278**, 2159–2164 (2011). 10.1098/rspb.2010.2342 PMC3107632 21159680

[10] S. Mathew, R. Boyd, The cost of cowardice: Punitive sentiments towards free riders in Turkana raids. Evol. Hum. Behav. **35**, 58–64 (2014).

[11] S. Mathew, R. Boyd, Punishment sustains large-scale cooperation in prestate warfare. Proc. Natl. Acad. Sci. U.S.A. **108**, 11375–11380 (2011). 21670285 10.1073/pnas.1105604108 PMC3136302

[12] P. Wiessner, Norm enforcement among the Ju/’hoansi bushmen: A case of strong reciprocity? Hum. Nat. **16**, 115–145 (2005). 26189619 10.1007/s12110-005-1000-9

[13] E. Vriens, G. Andrighetto, L. Tummolini, Risk, sanctions and norm change: The formation and decay of social distancing norms. Philos. Trans. R. Soc. B **379**, 20230035 (2024). 10.1098/rstb.2023.0035 PMC10799734 38244600

[14] E. O. Asekun-Olarinmoye, O. A. Amusan, The impact of health education on attitudes towards female genital mutilation (FGM) in a rural Nigerian community. Eur. J. Contracept. Reprod. Health Care **13**, 289–297 (2008). 18609348 10.1080/13625180802075174

[15] S. Vogt, N. A. Mohmmed Zaid, H. El Fadil Ahmed, E. Fehr, C. Efferson, Changing cultural attitudes towards female genital cutting. Nature **538**, 506–509 (2016). 27732586 10.1038/nature20100

[16] B. Shell-Duncan, K. Wander, Y. Hernlund, A. Moreau, Dynamics of change in the practice of female genital cutting in senegambia: Testing predictions of social convention theory. Soc. Sci. Med. **73**, 1275–1283 (2011). 21920652 10.1016/j.socscimed.2011.07.022 PMC3962676

[17] N. Belle, P. Cantarelli, Nudging public employees through descriptive social norms in healthcare organizations. Public Adm. Rev. **81**, 589–598 (2021).

[18] M. Bergquist, A. Nilsson, The dos and don’ts in social norms: A descriptive don’t-norm increases conformity. J. Theor. So. Psychol. **3**, 158–166 (2019).

[19] S. E. Bokemper , Experimental evidence that changing beliefs about mask efficacy and social norms increase mask wearing for Covid-19 risk reduction: Results from the united states and Italy. PLoS One **16**, e0258282 (2021). 34634089 10.1371/journal.pone.0258282 PMC8504748

[20] N. Bardsley, R. Sausgruber, Conformity and reciprocity in public good provision. J. Econ. Psychol. **26**, 664–681 (2005).

[21] J. Shang, R. Croson, A field experiment in charitable contribution: The impact of social information on the voluntary provision of public goods. Econ. Pol. **119**, 1422–1439 (2009).

[22] E. L. Krupka, R. A. Weber, Identifying social norms using coordination games: Why does dictator game sharing vary? J. Eur. Econ. Assoc. **11**, 495–524 (2013).

[23] C. S. Crandall, S. Eidelman, L. J. Skitka, G. S. Morgan, Status quo framing increases support for torture. Soc. Influ. **4**, 1–10 (2009).

[24] S. Eidelman, C. S. Crandall, J. Pattershall, The existence bias. J. Pers. Soc. Psychol. **97**, 765 (2009). 19857000 10.1037/a0017058

[25] M. Bucholtz, “Word up: Social meanings of slang in California youth culture” in A Cultural Approach to Interpersonal Communication: Essential Readings, L. Monaghan, J. E. Goodman, J. Robinson, Eds. (Wiley-Blackwell, 2012), pp. 274–297.

[26] V. Capraro, D. G. Rand, Do the right thing: Experimental evidence that preferences for moral behavior, rather than equity or efficiency per se, drive human prosociality. Judgm. Decis. Mak. **13**, 99–111 (2018).

[27] C. Bicchieri, E. Xiao, Do the right thing: But only if others do so. J. Behav. Decis. Mak. **22**, 191–208 (2009).

[28] S. L. Heiman , Descriptive norms caused increases in mask wearing during the Covid-19 pandemic. Sci. Rep. **13**, 11856 (2023). 37481635 10.1038/s41598-023-38593-w PMC10363160

[29] G. Andrighetto, D. Grieco, L. Tummolini, Perceived legitimacy of normative expectations motivates compliance with social norms when nobody is watching. Front. Psychol. **6**, 1413 (2015). 26500568 10.3389/fpsyg.2015.01413 PMC4593938

[30] A. Szekely , Evidence from a long-term experiment that collective risks change social norms and promote cooperation. Nat. Commun. **12**, 5452 (2021). 34526490 10.1038/s41467-021-25734-w PMC8443614

[31] G. A. Akerlof, R. E. Kranton, Economics and identity. Q. J. Econ. **115**, 715–753 (2000).

[32] T. Kuran, W. H. Sandholm, Cultural integration and its discontents. Rev. Econ. Stud. **75**, 201–228 (2008).

[33] L. Sacconi, M. Faillo, Conformity, reciprocity and the sense of justice. How social contract-based preferences and beliefs explain norm compliance: The experimental evidence. Const. Polit. Econ. **21**, 171–201 (2010).

[34] R. Golman, G. Loewenstein, K. O. Moene, L. Zarri, The preference for belief consonance. J. Econ. Perspect. **30**, 165–188 (2016).

[35] J. Realpe-Gómez, G. Andrighetto, L. G. Nardin, J. A. Montoya, Balancing selfishness and norm conformity can explain human behavior in large-scale prisoner’s dilemma games and can poise human groups near criticality. Phys. Rev. E **97**, 042321 (2018). 29758626 10.1103/PhysRevE.97.042321

[36] D. Tverskoi, A. Guido, G. Andrighetto, A. Sánchez, S. Gavrilets, Disentangling material, social, and cognitive determinants of human behavior and beliefs. Hum. Soc. Sci. Commun. **10**, 236 (2023).

[37] J. Guo, Wandering on the Edge- the Changes in a Frontier Minority Nationality Village During About 60 Years [Bianyuan de Youyi- yige Shaoshu minzu Cunzhuang jin 60 Nian Bianqian] (Kunming Yunnan People’s Publishing House [Yunnan Renmin Chubanshe], 2010).

[38] S. Gros, The bittersweet taste of rice. Sloping land conversion and the shifting livelihoods of the drung in northwest Yunnan, China. Himal. J. Assoc. Nepal Himal. Stud. **34**, 81–96 (2014).

[39] C. Bicchieri, Norms in the Wild: How to Diagnose, Measure, and Change Social Norms (Oxford University Press, Oxford, 2016), p. 288.

[40] T. Kuran, Private Truths, Public Lies: The Social Consequences of Preference Falsification (Harvard University Press, 1998).

[41] L. Bursztyn, A. L. González, D. Yanagizawa-Drott, Misperceived social norms: Women working outside the home in Saudi Arabia. Am. Econ. Rev. **110**, 2997–3029 (2020).

[42] D. T. Miller, B. Monin, D. A. Prentice, Pluralistic Ignorance and Inconsistency between Private Attitudes and Public Behaviors (Psychology Press, 1999), pp. 95–113.

[43] A. M. Ishungisa , What do other men think? understanding (mis) perceptions of peer gender role ideology among young Tanzanian men. J. Roy. Anthropol. Inst. (2024).

[44] P. Graeff, S. Sattler, G. Mehlkop, C. Sauer, Incentives and inhibitors of abusing academic positions: Analysing university students’ decisions about bribing academic staff. Eur. Sociol. Rev. **30**, 230–241 (2014).

[45] S. Sattler, C. Sauer, G. Mehlkop, P. Graeff, The rationale for consuming cognitive enhancement drugs in university students and teachers. PLoS One **8**, e68821 (2013). 23874778 10.1371/journal.pone.0068821 PMC3714277

[46] S. Sattler, P. Graeff, S. Willen, Explaining the decision to plagiarize: An empirical test of the interplay between rationality, norms, and opportunity. Deviant Behav. **34**, 444–463 (2013).

[47] K. D. Opp, “When do people follow norms and when do they pursue their interests” in Social Dilemmas, Institutions, and the Evolution of Cooperation, B. Jann, W. Przepiorka, Eds. (Berlin: de Gruyter, 2017), pp. 119–41.

[48] M. Czajkowski, N. Hanley, K. Nyborg, Social norms, morals and self-interest as determinants of pro-environment behaviours: The case of household recycling. Environ. Resour. Econ. **66**, 647–670 (2017).

[49] K. D. Opp, How do norms emerge? An outline of a theory Mind Soc. **2**, 101–128 (2001).

[50] A. Lindbeck, Incentives and social norms in household behavior. Am. Econ. Rev. **87**, 370–377 (1997).

[51] S. Mollen, R. N. Rimal, R. A. Ruiter, G. Kok, Healthy and unhealthy social norms and food selection. Findings from a field-experiment. Appetite **65**, 83–89 (2013). 23402712 10.1016/j.appet.2013.01.020

[52] L. Salmivaara, C. Lombardini, L. Lankoski, Examining social norms among other motives for sustainable food choice: The promise of descriptive norms. J. Clean. Prod. **311**, 127508 (2021).

[53] A. Lac, C. D. Donaldson, Testing competing models of injunctive and descriptive norms for proximal and distal reference groups on alcohol attitudes and behavior. Addict. Behav. **78**, 153–159 (2018). 29175291 10.1016/j.addbeh.2017.11.024

[54] H. W. Perkins, The Social Norms Approach to Preventing School and College Age Substance Abuse: A Handbook for Educators, Counselors, and Clinicians (John Wiley & Sons, 2003).

[55] H. Cho, Influences of norm proximity and norm types on binge and non-binge drinkers: Examining the under-examined aspects of social norms interventions on college campuses. J. Subst. Use **11**, 417–429 (2006).

[56] H. Allcott, Social norms and energy conservation. J. Public Econ. **95**, 1082–1095 (2011).

[57] N. J. Goldstein, R. B. Cialdini, V. Griskevicius, A room with a viewpoint: Using social norms to motivate environmental conservation in hotels. J. Consum. Res. **35**, 472–482 (2008).

[58] M. Hallsworth, J. A. List, R. D. Metcalfe, I. Vlaev, The behavioralist as tax collector: Using natural field experiments to enhance tax compliance. J. Public Econ. **148**, 14–31 (2017).

[59] N. Cheeseman, C. Peiffer, The curse of good intentions: Why anticorruption messaging can encourage bribery. Am. Polit. Sci. Rev. **116**, 1081–1095 (2022).

[60] A. Wismans , Hygiene and social distancing as distinct public health related behaviours among university students during the COVID-19 pandemic. Social Psychol. Bull. **15**, 4383 (2020).

[61] D. Martinez, C. Parilli, C. Scartascini, A. Simpser, Let’s (not) get together! The role of social norms on social distancing during COVID-19 PLoS One **16**, e0247454 (2021). 33651809 10.1371/journal.pone.0247454 PMC7924783

[62] R. B. Bird, B. Scelza, D. W. Bird, E. A. Smith, The hierarchy of virtue: Mutualism, altruism and signaling in martu women’s cooperative hunting. Evol. Hum. Behav. **33**, 64–78 (2012).

[63] J. Chapelle, F. Schütze, Black Nomads of the Sahara (Librairie Plon, 1957).

[64] M. S. Alvard, D. A. Nolin, Rousseau’s whale hunt? Coordination among big-game hunters Curr. Anthropol. **43**, 533–559 (2002).

[65] C. Engel, Dictator games: A meta study. Exp. Econ. **14**, 583–610 (2011).

[66] E. Fehr, K. M. Schmidt, A theory of fairness, competition, and cooperation. Q. J. Econ. **114**, 817–868 (1999).

[67] C. T. Dawes, J. H. Fowler, T. Johnson, R. McElreath, O. Smirnov, Egalitarian motives in humans. Nature **446**, 794–796 (2007). 17429399 10.1038/nature05651

[68] P. R. Blake, K. McAuliffe, ‘I had so much it didn’t seem fair’: Eight-year-olds reject two forms of inequity. Cognition **120**, 215–224 (2011). 21616483 10.1016/j.cognition.2011.04.006

[69] P. R. Blake , The ontogeny of fairness in seven societies. Nature **528**, 258–261 (2015). 26580018 10.1038/nature15703

[70] J. Ulber, K. Hamann, M. Tomasello, Young children, but not chimpanzees, are averse to disadvantageous and advantageous inequities. J. Exp. Child Psychol. **155**, 48–66 (2017). 27918977 10.1016/j.jecp.2016.10.013

[71] T. C. Schelling, The Strategy of Conflict (Harvard University Press, 1990).

[72] J. Mehta, C. Starmer, R. Sugden, “An experimental investigation of focal points in coordination and bargaining: Some preliminary results” in Decision Making Under Risk and Uncertainty. Theory and Decision Library, J. Geweke, Ed. (Springer, Dordrecht, 1992), vol. 22.

[73] H. P. Young, An evolutionary model of bargaining. J. Econ. Theory **59**, 145–168 (1993).

[74] R. Sugden, The Economics of Rights, Co-operation and Welfare (Palgrave Macmillan UK, 2005). http://link.springer.com/10.1057/9780230536791.

[75] R. Boyd, S. Mathew, Arbitration supports reciprocity when there are frequent perception errors. Nat. Hum. Behav. **5**, 596–603 (2021). 33398142 10.1038/s41562-020-01008-1

[76] B. Lindström, S. Jangard, I. Selbing, A. Olsson, The role of a “common is moral’’ heuristic in the stability and change of moral norms. J. Exp. Psychol. Gen. **147**, 228 (2018). 28891657 10.1037/xge0000365

[77] K. Eriksson, P. Strimling, J. C. Coultas, Bidirectional associations between descriptive and injunctive norms. Organ. Behav. Hum. Decis. Process. **129**, 59–69 (2015).

[78] G. Brennan, L. Eriksson, R. E. Goodin, N. Southwood, Explaining Norms (Oxford University Press, USA, 2013).

[79] C. Bicchieri, E. Dimant, S. Gächter, D. Nosenzo, Social proximity and the erosion of norm compliance. Games Econom. Behav. **132**, 59–72 (2022).

[80] M. J. Gelfand, J. R. Harrington, The motivational force of descriptive norms: For whom and when are descriptive norms most predictive of behavior? J. Cross Cult. Psychol. **46**, 1273–1278 (2015).

[81] J. Henrich, The evolution of costly displays, cooperation and religion: Credibility enhancing displays and their implications for cultural evolution. Evol. Hum. Behav. **30**, 244–260 (2009).

[82] J. A. Everett, L. Caviola, G. Kahane, J. Savulescu, N. S. Faber, Doing good by doing nothing? The role of social norms in explaining default effects in altruistic contexts Eur. J. Soc. Psychol. **45**, 230–241 (2015).

[83] C. R. Mortensen , Trending norms: A lever for encouraging behaviors performed by the minority. Soc. Psychol. Person. Sci. **10**, 201–210 (2019).

[84] C. Efferson, S. Vogt, A. Elhadi, H. E. F. Ahmed, E. Fehr, Female genital cutting is not a social coordination norm. Science **349**, 1446–1447 (2015). 26404811 10.1126/science.aaa7978

[85] C. Efferson, S. Vogt, E. Fehr, The promise and the peril of using social influence to reverse harmful traditions. Nat. Hum. Behav. **4**, 55–68 (2020). 31792402 10.1038/s41562-019-0768-2

[86] R. H. Thaler, Misbehaving: The Making of Behavioral Economics (W. W. Norton & Company, New York, 2016).

[87] N. Nikiforakis, D. Engelmann, Altruistic punishment and the threat of feuds. J. Econ. Behav. Organ. **78**, 319–332 (2011).

[88] N. J. Raihani, R. Bshary, Punishment: One tool, many uses. Evol. Human Sci. **1**, e12 (2019). 37588410 10.1017/ehs.2019.12 PMC10427336

[89] A. Dreber, D. G. Rand, D. Fudenberg, M. A. Nowak, Winners don’t punish. Nature **452**, 348–351 (2008). 18354481 10.1038/nature06723 PMC2292414

[90] M. A. Nowak, K. Sigmund, Evolution of indirect reciprocity by image scoring. Nature **393**, 573–577 (1998). 9634232 10.1038/31225

[91] A. Leibbrandt, R. López-Pérez, The dark side of altruistic third-party punishment. J. Confl. Resolut. **55**, 761–784 (2011).

[92] J. Henrich, R. Boyd, Why people punish defectors: Weak conformist transmission can stabilize costly enforcement of norms in cooperative dilemmas. J. Theor. Biol. **208**, 79–89 (2001). 11162054 10.1006/jtbi.2000.2202

[93] A. Falk, E. Fehr, U. Fischbacher, Driving forces behind informal sanctions. Econometrica **73**, 2017–2030 (2005).

[94] J. J. Jordan, D. G. Rand, Third-party punishment as a costly signal of high continuation probabilities in repeated games. J. Theor. Biol. **421**, 189–202 (2017). 28390842 10.1016/j.jtbi.2017.04.004

[95] J. J. Jordan, D. G. Rand, Signaling when no one is watching: A reputation heuristics account of outrage and punishment in one-shot anonymous interactions. J. Pers. Soc. Psychol. **118**, 57 (2020). 30985155 10.1037/pspi0000186

[96] R. Boyd, H. Gintis, S. Bowles, Coordinated punishment of defectors sustains cooperation and can proliferate when rare. Science **328**, 617–620 (2010). 20431013 10.1126/science.1183665

[97] S. Mathew, How the second-order free rider problem is solved in a small-scale society. Am. Econ. Rev. **107**, 578–581 (2017).

[98] L. Molleman, F. Kölle, C. Starmer, S. Gächter, People prefer coordinated punishment in cooperative interactions. Nat. Hum. Behav. **3**, 1145–1153 (2019). 31477909 10.1038/s41562-019-0707-2

[99] M. Yan, Z. Li, Y. Li, R. Boyd, S. Mathew, Data and meta data for “A norm about harvest division is maintained by a desire to follow tradition, not by social policing.” GitHub. https://github.com/Minhua-Yan/norm-tradition. Deposited 6 April 2024. 10.1073/pnas.2413214122 PMC12207420 40540591

