# Supplementary material for: A norm about harvest division is maintained by a desire to follow tradition, not by social policing
Source: Proc Natl Acad Sci U S A. 2025 Jun 20;122(25):e2413214122. doi: 10.1073/pnas.2413214122 (PMC12207420; doi:10.1073/pnas.2413214122)
Supplement: Supplementary file 1 — Appendix 01 (PDF) [file pnas.2413214122.sapp.pdf]

Supporting Information for

A norm about harvest division is maintained by a desire to follow tradition, not by social policing

Minhua Yan, Zhizhong Li, Yuanmei Li, Robert Boyd and Sarah Mathew

Minhua Yan
E-mail: minhua.yan@iast.fr

This PDF file includes:

Supporting text
Figs. S1 to S2
Table S1
SI References

Supporting Information Text

Appendix A: A map of Derung communities' geographical spread

Map 1. The geographical distribution of Derung (Rawang) people in China and Myanmar.

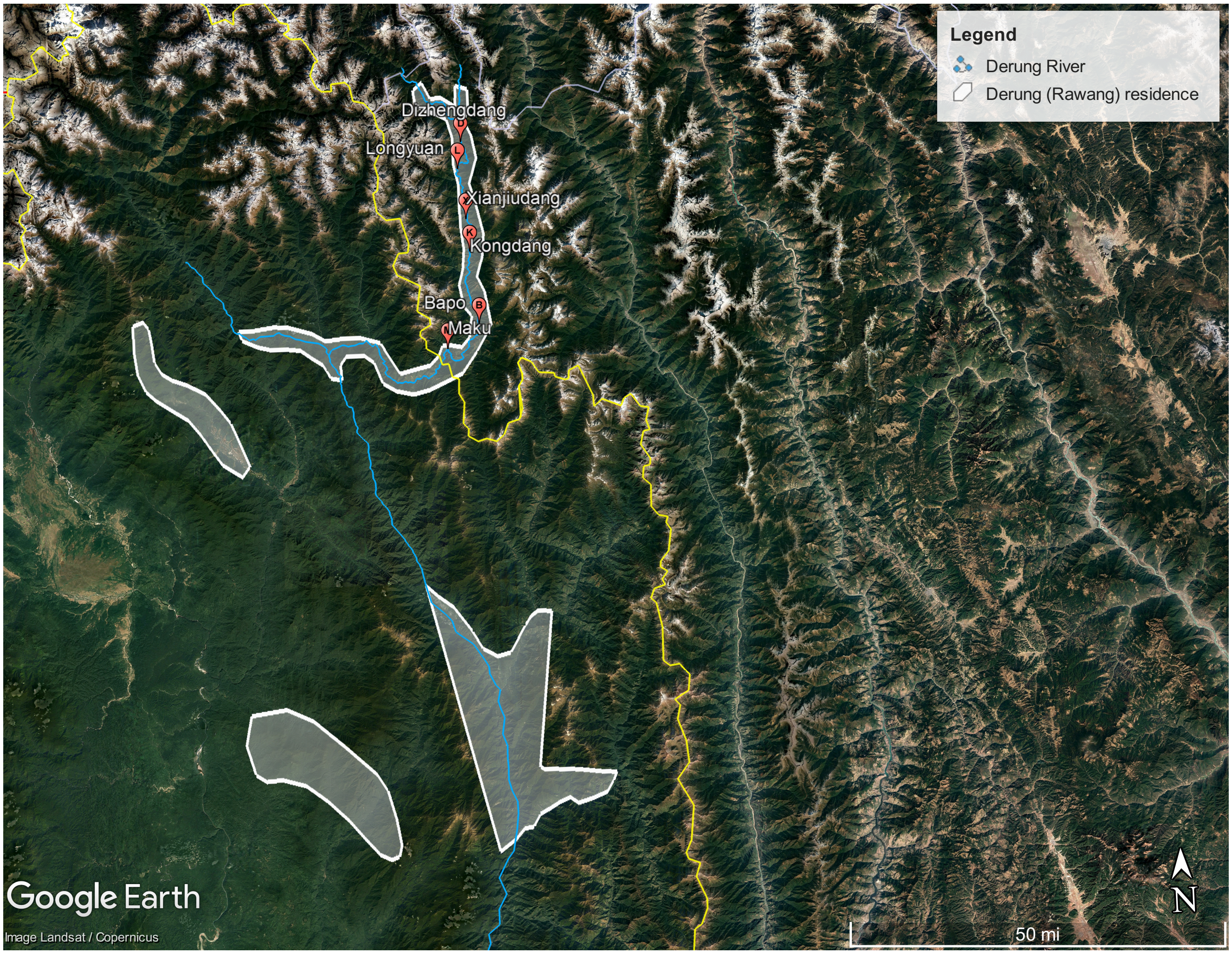

Appendix B: The recruitment and consent script for the interview on real-life co-farming partnerships

My name is Yan Minhua and I am a student at Arizona State University. I am here to conduct research. Li Yuanmei/Li Zhizhong is responsible for the translation. We would like to invite you to participate in an interview about your farming. All farming issue deciders in Dizhengdang are invited. The interview takes 30-120 minutes. If you participate, you will receive cash compensation based on the time you spent on the interview. The compensation rate is 40 CNY per hour. During the interview, I will ask you which plots of land you farm on, what you farm on each plot, how much is harvested, and whether it is co-farmed with others. For co-farmed plots, I will ask for your opinion on the co-farming. You can withdraw at any time during the interview. You can also skip questions that you don't want to answer. No matter whether you withdraw or skip questions, you will receive compensation based on the time spent.

Your answer will not be disclosed by the research team to other villagers or the government. The data collected from this interview will be published in academic journals. Your identity is confidential. Only your informed consent process will be recorded. These recordings will not be disclosed to anyone other than to the research ethics committee if necessary. If I want to take photos of you participating in the research, I will ask for your additional consent.

If you have any questions, concerns, or complaints, you can inform Li Zhizhong, Li Yuanmei, or the head of the research team, Yan Minhua. (Phone number: 13522382042)

Appendix C: A semi-structured interview script from one study participant who was interviewed in November, 2020

General Farming Conditions

Q1. Do you have just enough subsistence crop harvests (e.g., potatoes, corn) for your household, more than enough that you cannot consume them all before they rot, or not enough subsistence crop harvests?
A1: There is not enough corn, because need to feed chickens and pigs. There are more potatoes harvested than we can eat.
Q2. Do you feel your household is in shortage of land (farming land or forest land)? (If subject answered yes) How much more would you like to have? In what location?
A2: There is no shortage of fixed land, and there is no shortage of forest land.
Q3. Is subsistence crop farming important to your household? (If you switch to farming all cash crops like RHIZOMA PARIDIS instead and purchasing your grains, will you like it better, like is less, or feel indifferent?)
A3: It is better to grow a little subsistence crops. If (we) don't farm, there is nothing to eat. As for buying food, you may not be able to buy it at all times.
Q4. Is farming important to your household? (If you quit farming and switch to earning incomes by going outside to work waged jobs or accommodating tourists, will you like it better, like it less, or feel indifferent?)
A4: It is better to farm a bit of land. The store is mainly for convenience (instead of relying on it as the income source).
Q5. Do you feel your household has enough labor for farming?
A5: There is a shortage. There are only two laborers in my household. It would be enough if there are 2 more.

Current Co-farming

Q6. Who are you co-farming with? What plants are your co-farming?
A6: 1) LD190718: SB38624's land in LM pasture, about 1 mu, SB38624 and SB72049 (SB56721's husband) two households co-farming corn and potato; 2) LD838648: Gongqian communal land, 1 Mu, SB56721 and SB74798 two households co-farming potatoes and corn;
Q7. When did you first start this co-farming partnership? Have you been farming together every year since then?
A7: 2) 2019
Q8. Why are you farming cooperatively with others?
A8: 2) Because of the lack of labor force in my own household. At the beginning it is difficult to dig the land. It took a week to dig out the stones. At that time, SB74798's household, SB74798 and SB50131 two laborers went together; for SB56721's household, the SB56721 couple went.
Q9. Who proposed to co-farm? (If subject) Why did you select this partner?
A9: 2) Proposed by SB56721. SB74798 has less land, and those with more land may not have the time to CF with us.
Q10. How are you benefiting from this co-farming?
Q11. How many laborers does each family provide? Who provides the land? Who provides how much seeds, fertilizers, land membranes, etc.?
A11: 2) In 2019, the seeds were contributed by the two households together, and both contributed the same amount. Which household contributed the land membranes and the fertilizers depended on which household had them, because we are all family. The deceased husband of SB74798 and SB72049's father are full brothers. In 2019, the SB56721 household always went 2 people, and the SB74798 family went 2 people. In 2020, each household contributed about 5 Jin of land membranes, and each household contributed about 15 Jin of chemical fertilizers. In 2020, the SB56721 household went 2 people every time. The SB74798 household, SB74798 went every time, SB73069 and SB37757 went to help every time. SB50131 (SB74798's son) and his wife also went during planting; To return the favor of SB73069 and SB37757, the SB56721 couple went when digging potatoes. The other times, SB74798 returned the favor by herself. Overall, the SB74798 household worked more, because they had more helpers, and our household (the laborers were) only us couple.
Q12. How much of each kind of crop did you harvest? How much of each kind of crop did each family get in division?
A12: 2) There are 5 baskets of mature corn, 1 basket of fresh corn. Each household got divided 2 baskets of mature corn. One basket was made into corn flakes and then divided equally, each household got about 10 Jin (of corn flakes). Other than what the two co-farming households got, gave the SB73069 household 5 Jin of corn flakes, and gave the SB37757 household 5 Jin of corn flakes. Only gave the helpers corn flakes, not corn on cobs. Each co-farming household received 2 baskets of potatoes, did not gift to helpers, because all households have potatoes, no need to gift it.
Q13. Did you and your partner(s) discuss how you would divide before you decided to co-farm? Did you and your partner(s) discuss which household should contribute how many laborers, or what counts as a household? How did you discuss it? (If household is defined differently from residential pattern) Why did you define a household in this way?
A13: 2) No discussion before or after harvest; No discussion about how many labors each household would contribute.
Q14. Has the division rule changed during the years of your co-farming with this partner?
Q15. Are there alternative ways of division that you think are more reasonable? (Will you like it better if you and your current co-farming partners track attendance and divide in proportion to labor contribution? Why?/Why not?)
A15: 2) It is more reasonable for SB74798's household to get divided more for contributing more laborers, I proposed but SB74798 did not agree.

Q16. In this year or past years, have you ever felt your household is not making as much contribution to the co-farming as your partner(s)? (If subject answered yes) Is that an issue to you? Have you proposed ways to make up for that?

Q17. Has/Have your partner(s) ever complained about your household not making enough contribution?

Q18. In this year or past years, have you ever felt the other household(s) is/are not making as much contribution as your household? (If subject answered yes) Have you told the other household? Have you or the other household proposed ways to make up for that?

Q19. Have you ever complained to others about your co-farming partner(s)' household(s) not making enough contribution?

Q20. If you propose (alternative division), how do you think your partner will respond behaviorally and emotionally? (Will they get upset? Will they complain about your behavior to other people? Is it possible for them to end the co-farming relationship with you because of this?)

Q21. If other people in the village hear about your above behavior, how do you think they will react to that behaviorally and emotionally? (Will they think you are petty, selfish, or bad-hearted? Is it possible for they to be unwilling to co-farm with you in the future when you wish to co-farm with them?)

Q22. If your partner proposes (alternative division), how will you react to that behaviorally and emotionally? (Will you get upset? Will you complain to others? Is it possible for you to end the co-farming relationship with them because of this?)

Co-farming Notions Rejected

Q23. Has it ever happened that you proposed to farm together with others but they did not agree? List as many of such cases as you can remember. (If yes, for each such case ask the following set of questions: When did it happen? Who was it that you asked to co-farm with? Why did you want to co-farm with them? What was the reason they gave you for not being able to co-farm with you?)

A23: No.

Q24. Has it ever happened that other people proposed to co-farm with you but you could not agree? List as many of such cases as you can remember. (If yes, for each such case ask the following set of questions: When did it happen? Who proposed it? Why couldn't you co-farm with them? (If the subject rejected some but accepted some others) Why did you agree to co-farm with (some other's name) but not the above mentioned people?)

A24: No.

Past Co-farming

Q25. Did you participate in co-farming that happened in the past and has already ended? Was there co-farming that ended before any harvest? (If yes, for each such case ask the following set of questions: Who did you co-farm with? When did it start? When did it end? What did you farm? Who provided the land? What was the area of the land? How many laborers did each household provide? How many seeds, fertilizers, membranes did each household provide? How did you divide each kind of crop you farmed together? Why did the co-farming partnership stop?)

A25: No.

Future Co-farming

Q26. Do you have the intention to do (more) co-farming? (It could be subsistence crops or cash crops, on farming land or forest land)

A26: No, there are only two laborers in my family and we cannot finish that much work.

Q27. (If answered yes in Q26) What kind of partner do you want? (number of laborers, area of land, land position, personality, economic capability, etc.?)

Normative Knowledge

Q28. Do you know how other households in village D divide when they farm subsistence crops? If so, how do they divide?

A28: Don't know. It is probably divided equally; even if the labor, land, and seed contribution are not the same, it is still divided equally, because (the people in village D) are not so calculating.

Q29. Do you know how other households in village D divide when they farm cash crops? If so, how do they divide?

A29: The co-farming of cash crops may be divided in proportion to the number of labors. If it is not organized by the government, but the people themselves want to co-farm, it may be divided equally, no matter who contributes more labor and land.

Appendix D: Derung subjects guessed the majority preference for the best offer in the ultimatum game 2:1 treatment based on their own preference.

How did the Derung participants guess the majority preference? There are three forms of information they may base the guess on: 1) the common behavior in real-life co-farming, 2) the participant's own preference, and 3) others' expressed preferences for divisions in co-farming situations similar to the UG 2:1 context. The source of the participants' guesses could affect how confident they are about their guesses. Specifically, the subjects may feel more confident if the primary information source was the expressed preferences of other villagers, since it is a more direct form of information, while they may guess the same

majority preference but be more reluctant to use such guess to direct their behaviors if they have based the guess on their own preference and are less confident about its accuracy.

To distinguish among these three information sources and thus infer the participants' confidence in their guesses, we estimate the following statistical model for q, the probability that a subject guessed rewarding labor contribution as the majority preference. We approximate a participant's information about others' expressed preferences as her co-farming partners' stated preferences in the post-UG survey.

$$logit(q) = \alpha + \beta s + \gamma o$$

$q \in [0, 1]$ is the probability that a participant guessed most other villagers would prefer a division that rewarded labor contribution. q is predicted by three parameters— 1) α: a parameter capturing information shared by all participants, including that the common division in real-life co-farming is "HD"; 2) s: the participant's own preferred division, equalling 0 if the the participant's own preferred division is "HD" and 1 if own preferred division is "rewarding labor"; and 3) o: the difference between the proportion of those who preferred rewarding labor contribution and the proportion of those who preferred HD among the participants' co-farming partners in 2018 and 2019. In this dataset, the participants had 0 to 5 partners. The possible values of o in this dataset are $\{-1, -\frac{1}{3}, 0, \frac{1}{3}, \frac{1}{2}, 1\}$. $o = -1$ means all the participants' co-farming partners who participated in the post-UG survey preferred HD. $o = \frac{1}{3}$ happens when two thirds of the partners who participated in the post-UG survey preferred "rewarding labor" and one third preferred "HD". If one or more co-farming partner(s) of a participant did not participate in the post-UG survey, they are omitted in the calculation of o. If a participant did not participate in real-life co-farming and thus had no co-farming partner, or if none of their co-farming partners participated in the post-UG survey, their o is assigned the value 0. 12 out of the 72 participants had co-farming partners who did not participate in the post-UG survey; for one participant it was their only co-farming partner. 8 out of the 72 participants did not conduct co-farming in 2018 or 2019.

A Bayesian model (1) with nonrestrictive priors ($\alpha \sim N(\mu = 0, \sigma^2 = 2)$, $\beta \sim N(\mu = 0, \sigma^2 = 0.5)$, and $\gamma \sim N(\mu = 0, \sigma^2 = 0.5)$) (see Fig. S1) produces the estimates in Table S1. The biological meanings of the coefficients themselves are hard to interpret, but the "mean average partial effect" and "average partial effect 90% confidence interval" columns indicate how switching from $s = 1$ to $s = 2$ (i.e., for a subject to prefer a division that rewards labor vs. HD) for β or from $o = -0.5$ to $o = 0.5$ (i.e., switching from 75% to 25% of a subject's co-farming partners to prefer a division that rewards labor) for γ impacts q, averaged across the sample for the other parameter. The average partial effect of s going from 1 to 2 is expected to be 40.2%, meaning that when a subject's own preference changes from HD to "rewarding labor", her probability of guessing "rewarding labor" as the majority preference is expected to increase 40.2%. On the other hand, the average partial effect of o going from -0.5 to 0.5 is expected to be 4.8% and has a 90% confidence interval that includes 0, suggesting that when a subjects' co-farming partners' preference composition changes from 25% preferring "rewarding labor" to 75% preferring "rewarding labor", the probability of the subject guessing "rewarding labor" as the majority preference is expected to decrease 4.8% but may stay the same. Fig. S2 illustrates this result graphically. In conclusion, instead of believing the "public lie", subjects projected their own "private truth" onto others when guessing the population majority preference.

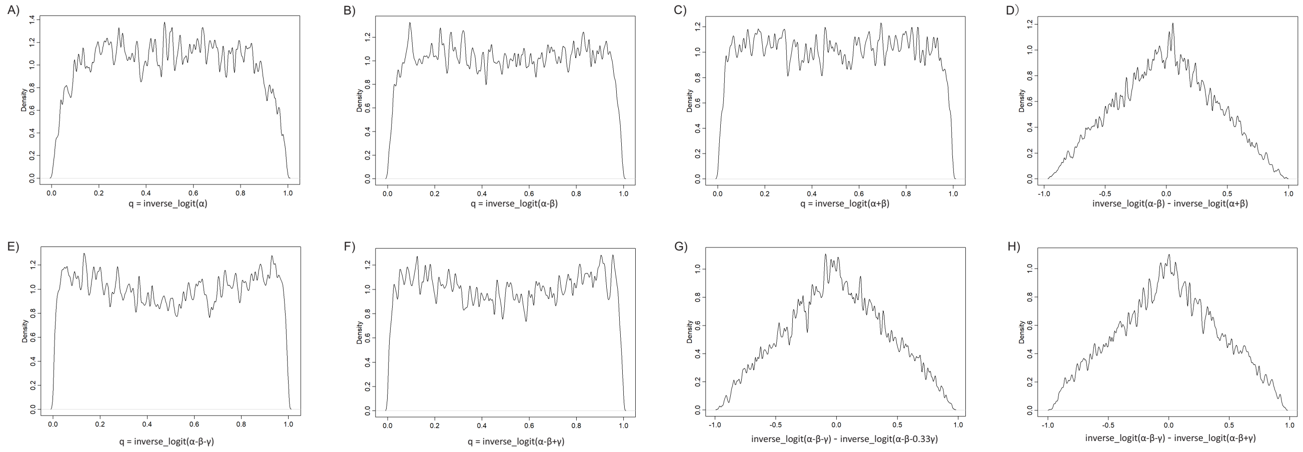

Fig. S1. Distribution of q, the predicted probability for a subject to guess "rewarding labor" as the majority preference given the priors $\alpha \sim N(\mu = 0, \sigma^2 = 2)$, $\beta \sim N(\mu = 0, \sigma^2 = 0.5)$. The priors are non-restrictive because all probabilities are roughly evenly distributed between 0 and 1, and all probability differences are centered around 0 but allows for all values between -1 and 1. A): Distribution of q from $logit(q) = \alpha$, i.e., when the only information is that shared by all participants, like that the common division in real-life co-farming is "HD"; B): Distribution of q from $logit(q) = \alpha + \beta s$, with $s = -1$, i.e., when in addition to shared information, the subject also considers her own preference of "HD"; C): Distribution of q from $logit(q) = \alpha + \beta s$, with $s = 1$, i.e., own preference is "rewarding labor"; D) Distribution of $q_1 - q_2$, where $logit(q_1) = \alpha + \beta s_1$ with $s_1 = -1$, $logit(q_2) = \alpha + \beta s_2$ with $s_2 = 1$, i.e., the difference in the probabilities of guessing 'rewarding labor" between non-co-farming subjects with different own preferences; E): Distribution of q from $logit(q) = \alpha + \beta s + \gamma o$, with $s = -1$ and $o = -1$, i.e., when own preference is "HD" and all co-farming partners prefer "HD". F): Distribution of q from $logit(q) = \alpha + \beta s + \gamma o$, with $s = -1$ and $o = 1$, i.e., when own preference is "HD" but all co-farming partners prefer "rewarding labor"; G) Distribution of $q_1 - q_2$, where $logit(q_1) = \alpha + \beta s + \gamma o_1$ with $s = -1$ and $o_1 = -1$, $logit(q_2) = \alpha + \beta s + \gamma o_2$ with $s = -1$ and $o_2 = -0.33$; H) Distribution of $q_1 - q_3$, where $logit(q_1) = \alpha + \beta s + \gamma o_1$ with $s = -1$ and $o_1 = -1$, $logit(q_2) = \alpha + \beta s + \gamma o_2$ with $s = -1$ and $o_2 = 1$;

Table S1. Model estimates

	coefficient mean	coefficient standard deviation	average partial effect mean across posterior	average partial effect 90% confidence interval
α	-0.67	0.47	—	—
β	1.55	0.36	54.9%	[33.2%, 73.3%]
γ	-0.20	0.52	-1.1%	[-10.1%, 8.1%]

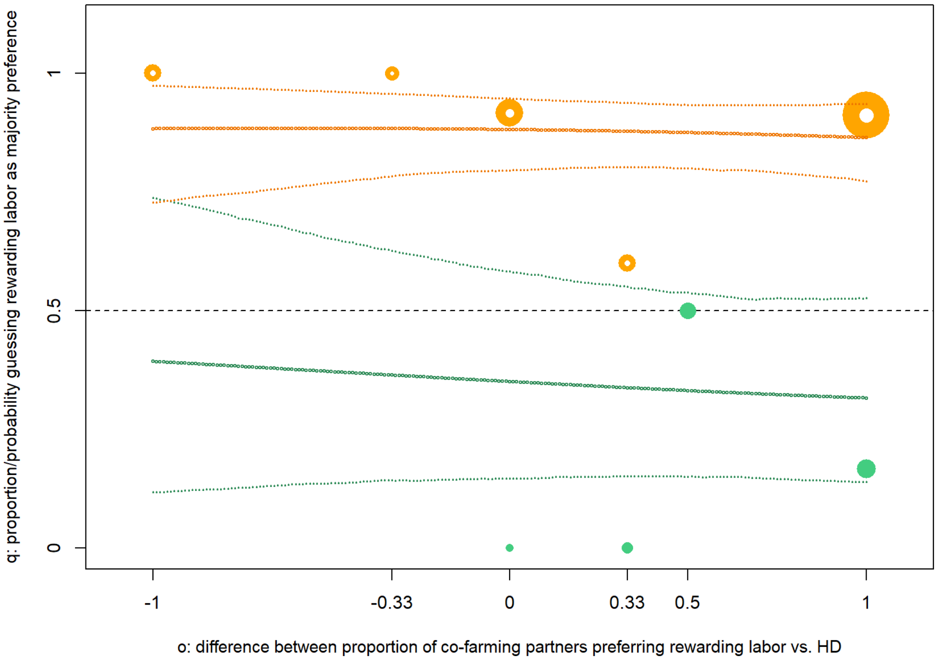

Fig. S2. Proportion of participants guessing "rewarding labor" as the majority preference (big dots) and model predictions (thick dotted lines, with thin dotted lines showing the 90% confidence interval boundaries) for the probability of a subject guessing "rewarding labor", separated by self preference (orange for "rewarding labor" and green for "HD") and co-farming partner preference distribution (horizontal axis). The model concludes that a subject's guess of majority preference is mainly determined by her own preference. Orange dots have white at the center to differentiate from green dots. The dots' area indicates the number of subjects with a self preference and co-farming partner preference distribution combination, with the smallest green dot at $o = 0.5$, $q = 0$ indicating 1 subject. The confidence interval (area between the two thin orange dotted lines) for when a subject preferred "rewarding labor" and the confidence interval (area between the two thin green dotted lines) for when a subject preferred "HD" (mostly) do not overlap. This means that the model believes subjects with different preferences would make different majority preference guesses given any co-farming partners' preference composition. The lines are mostly flat, suggesting that the model believes a subject's co-farming partner composition has minimal impact on their guesses of majority preference.

References

1. R McElreath, *Statistical rethinking: A Bayesian course with examples in R and Stan.* (Chapman and Hall/CRC), (2018).
